# 514 Incidence, Manifestation, Treatment and Outcomes related to Adrenal Insufficiency in Adult Burn Patients

**DOI:** 10.1093/jbcr/irad045.111

**Published:** 2023-05-15

**Authors:** Ana Segura, Dominick Curry, Andrew Rainey, Susan Smith

**Affiliations:** Orlando Health, Orlando, Florida; Orlando Regional Medical Center, Orlando, Florida; University of Florida, College of Public Health and Health Professions, Orlando, Florida; Orlando Regional Medical Center, Orlando, Florida; Orlando Health, Orlando, Florida; Orlando Regional Medical Center, Orlando, Florida; University of Florida, College of Public Health and Health Professions, Orlando, Florida; Orlando Regional Medical Center, Orlando, Florida; Orlando Health, Orlando, Florida; Orlando Regional Medical Center, Orlando, Florida; University of Florida, College of Public Health and Health Professions, Orlando, Florida; Orlando Regional Medical Center, Orlando, Florida; Orlando Health, Orlando, Florida; Orlando Regional Medical Center, Orlando, Florida; University of Florida, College of Public Health and Health Professions, Orlando, Florida; Orlando Regional Medical Center, Orlando, Florida

## Abstract

**Introduction:**

In the setting of hypotension, serum cortisol levels are obtained to eliminate adrenal insufficiency as a contributing cause. Morbidity, prolonged length of intensive care unit and mechanical ventilator dependent days with higher mortality rates have been associated with adrenal insufficiency. The primary objective was to identify patient characteristics and treatment indications, approaches, and outcomes in adult burn – injured patients diagnosed with adrenal insufficiency.

**Methods:**

This is a single center, five-year retrospective medical record review of adult patients aged 18 years and older admitted to the burn service were included. The data was analyzed using simple and correlational statistics to describe categorical data. Logistic regression, One-way ANOVA, and univariate analyses were conducted in Statistical Analysis System (SAS)® v 9.4.

**Results:**

Sixty nine patients were included, 64% male, 51years old, with 27 % total body surface area, nearly half with smoke inhalation, and 32% mortality.

Duration of hydrocortisone therapy in days, total dose in milligrams, duration of wean in days, and number of times therapy reinitiated were found to be (unadjusted) significantly related to intensive care length of stay, p = < 0.001. Hydrocortisone total dose in milligrams was found to be significantly related with increased vasopressor hours, p = 0.00927, p = 0.0163.

**Conclusions:**

Hypotension, leading to shock, directly correlates with overall burn survivability. Functional adrenal insufficiency may result for shock and sepsis. Hypotension was the instigating factor for hydrocortisone therapy. Random cortisol levels were obtained in all patients included in the study, with an average of 14 mcg/dl which did not exclude adrenal insufficiency as a contributing cause.

**Applicability of Research to Practice:**

This exploratory research will assist with formalizing a list of triggers for initiation of adrenal replacement therapy. These results are foundational in development of outcomes resulting from treatment with varying doses of hydrocortisone and lengths of therapy.

